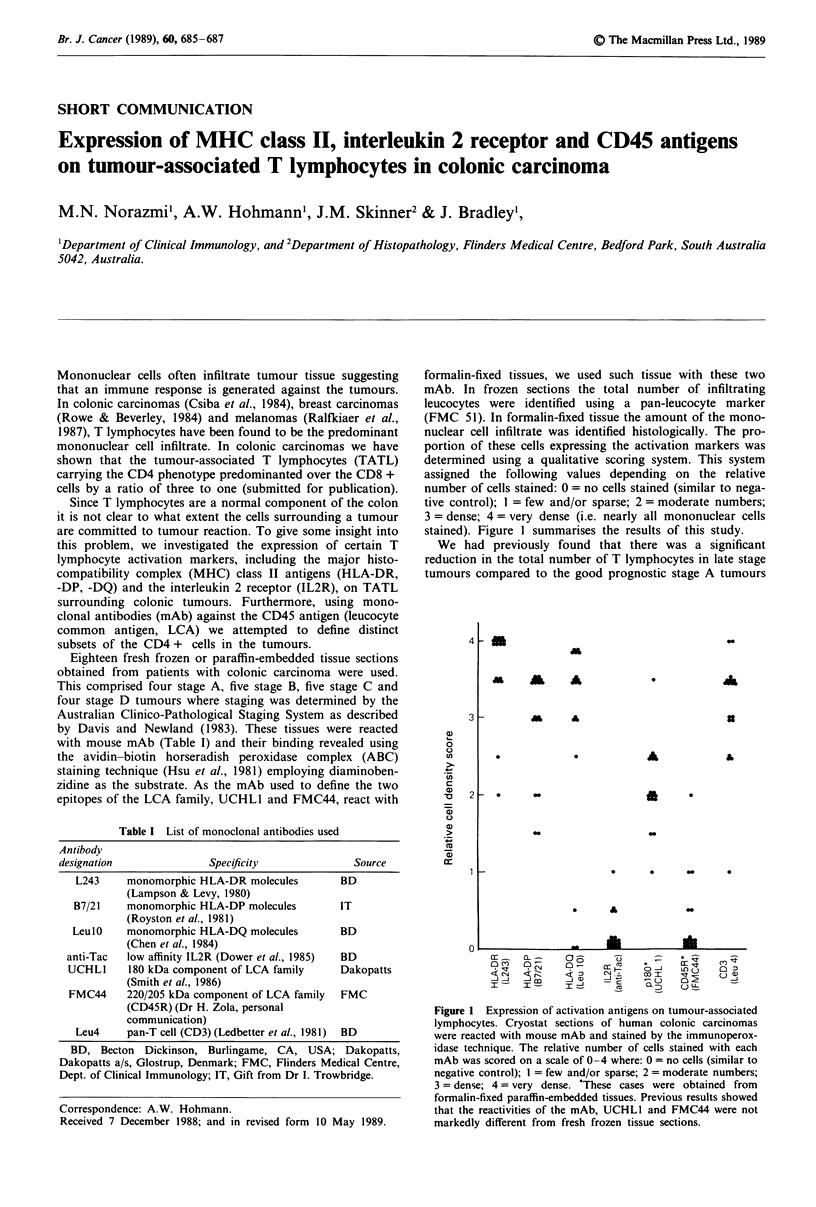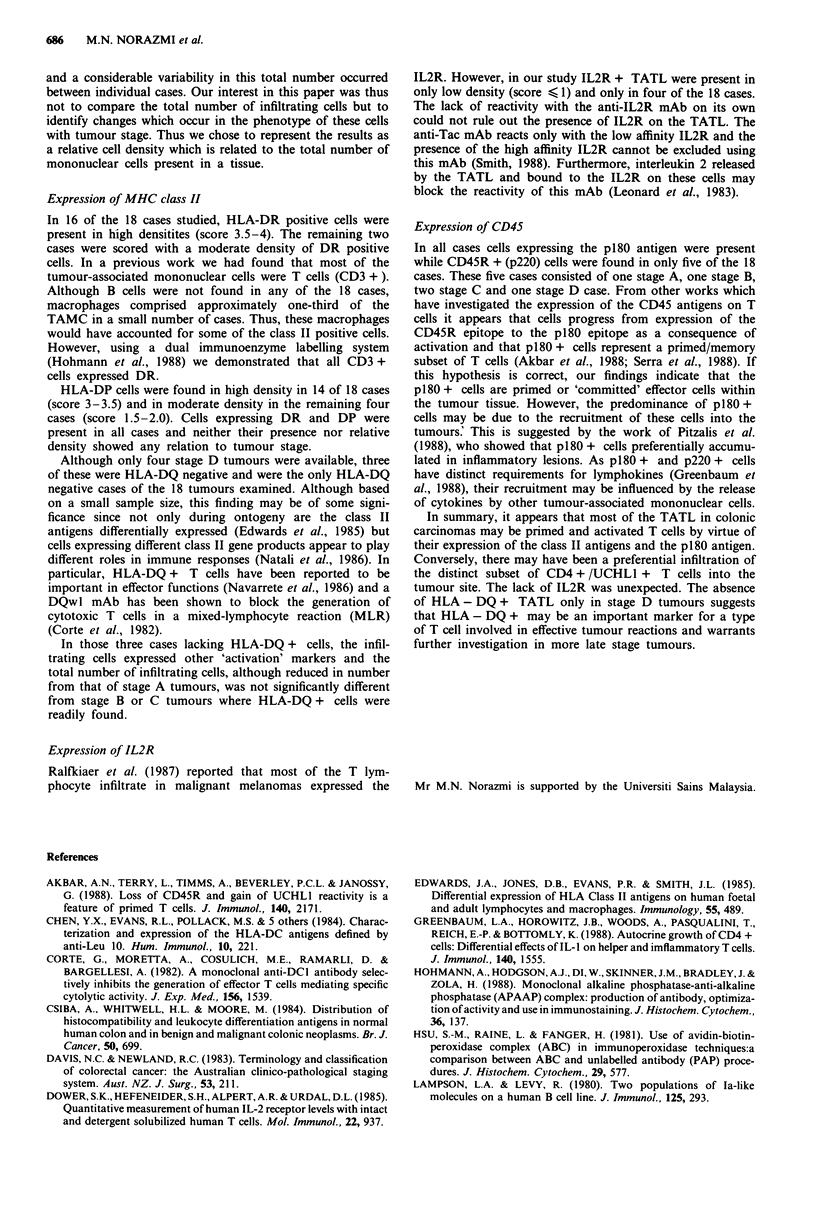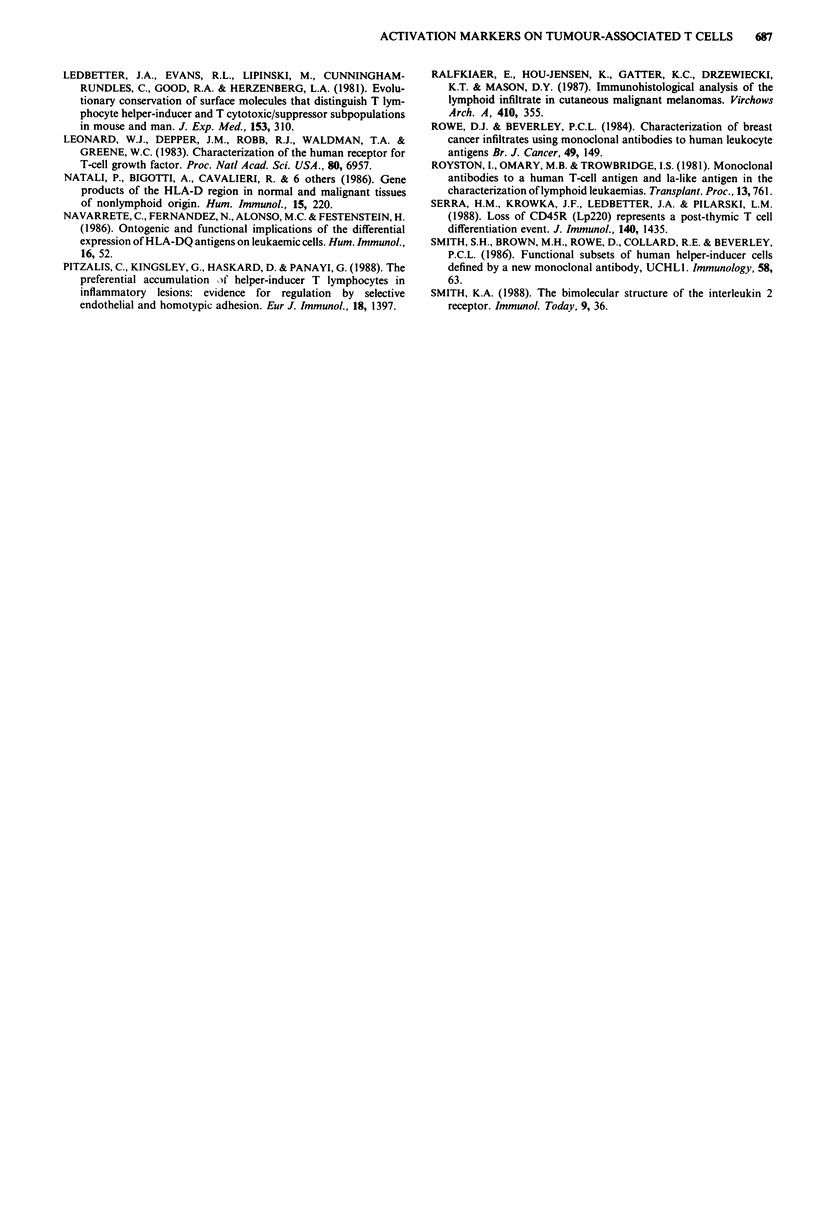# Expression of MHC class II, interleukin 2 receptor and CD45 antigens on tumour-associated T lymphocytes in colonic carcinoma.

**DOI:** 10.1038/bjc.1989.340

**Published:** 1989-11

**Authors:** M. N. Norazmi, A. W. Hohmann, J. M. Skinner, J. Bradley

**Affiliations:** Department of Clinical Immunology, Flinders Medical Centre, Bedford Park, South Australia.


*Br. J. Cancer* (1989), **60**, 685-687 © The Macmillan Press Ltd., 1989

SHORT COMMUNICATION

### Expression of MHC class II, interleukin 2 receptor and CD45 antigens on tumour-associated T lymphocytes in colonic carcinoma

M.N. Norazmi[1], A.W. Hohmann[1], J.M. Skinner[2] & J. Bradley[1],

[1]*Department of Clinical Immunology, and* [2]*Department of Histopathology, Flinders Medical Centre, Bedford Park, South Australia 5042, Australia.*

Mononuclear cells often infiltrate tumour tissue suggesting that an immune response is generated against the tumours. In colonic carcinomas (Csiba *et al.*, 1984), breast carcinomas (Rowe & Beverley, 1984) and melanomas (Ralfkiaer *et al.*, 1987), T lymphocytes have been found to be the predominant mononuclear cell infiltrate. In colonic carcinomas we have shown that the tumour-associated T lymphocytes (TATL) carrying the CD4 phenotype predominanted over the CD8 + cells by a ratio of three to one (submitted for publication).

Since T lymphocytes are a normal component of the colon it is not clear to what extent the cells surrounding a tumour are committed to tumour reaction. To give some insight into this problem, we investigated the expression of certain T lymphocyte activation markers, including the major histocompatibility complex (MHC) class II antigens (HLA-DR, -DP, -DQ) and the interleukin 2 receptor (IL2R), on TATL surrounding colonic tumours. Furthermore, using monoclonal antibodies (mAb) against the CD45 antigen (leucocyte common antigen, LCA) we attempted to define distinct subsets of the CD4 + cells in the tumours.

Eighteen fresh frozen or paraffin-embedded tissue sections obtained from patients with colonic carcinoma were used. This comprised four stage A, five stage B, five stage C and four stage D tumours where staging was determined by the Australian Clinico-Pathological Staging System as described by Davis and Newland (1983). These tissues were reacted with mouse mAb (Table I) and their binding revealed using the avidin-biotin horseradish peroxidase complex (ABC) staining technique (Hsu *et al.*, 1981) employing diaminobenzidine as the substrate. As the mAb used to define the two epitopes of the LCA family, UCHL1 and FMC44, react with

**Table I** List of monoclonal antibodies used

| *Antibody designation* | *Specificity* | *Source* |
|---|---|---|
| L243 | monomorphic HLA-DR molecules (Lampson & Levy, 1980) | BD |
| B7/21 | monomorphic HLA-DP molecules (Royston *et al.*, 1981) | IT |
| Leu10 | monomorphic HLA-DQ molecules (Chen *et al.*, 1984) | BD |
| anti-Tac | low affinity IL2R (Dower *et al.*, 1985) | BD |
| UCHL1 | 180 kDa component of LCA family (Smith *et al.*, 1986) | Dakopatts |
| FMC44 | 220/205 kDa component of LCA family (CD45R) (Dr H. Zola, personal communication) | FMC |
| Leu4 | pan-T cell (CD3) (Ledbetter *et al.*, 1981) | BD |

BD, Becton Dickinson, Burlingame, CA, USA; Dakopatts, Dakopatts a/s, Glostrup, Denmark; FMC, Flinders Medical Centre, Dept. of Clinical Immunology; IT, Gift from Dr I. Trowbridge.

Correspondence: A.W. Hohmann.
Received 7 December 1988; and in revised form 10 May 1989.

formalin-fixed tissues, we used such tissue with these two mAb. In frozen sections the total number of infiltrating leucocytes were identified using a pan-leucocyte marker (FMC 51). In formalin-fixed tissue the amount of the mononuclear cell infiltrate was identified histologically. The proportion of these cells expressing the activation markers was determined using a qualitative scoring system. This system assigned the following values depending on the relative number of cells stained: 0 = no cells stained (similar to negative control); 1 = few and/or sparse; 2 = moderate numbers; 3 = dense; 4 = very dense (i.e. nearly all mononuclear cells stained). Figure 1 summarises the results of this study.

We had previously found that there was a significant reduction in the total number of T lymphocytes in late stage tumours compared to the good prognostic stage A tumours

**Figure 1** Expression of activation antigens on tumour-associated lymphocytes. Cryostat sections of human colonic carcinomas were reacted with mouse mAb and stained by the immunoperoxidase technique. The relative number of cells stained with each mAb was scored on a scale of 0-4 where: 0 = no cells (similar to negative control); 1 = few and/or sparse; 2 = moderate numbers; 3 = dense; 4 = very dense. *These cases were obtained from formalin-fixed paraffin-embedded tissues. Previous results showed that the reactivities of the mAb, UCHL1 and FMC44 were not markedly different from fresh frozen tissue sections.

and a considerable variability in this total number occurred between individual cases. Our interest in this paper was thus not to compare the total number of infiltrating cells but to identify changes which occur in the phenotype of these cells with tumour stage. Thus we chose to represent the results as a relative cell density which is related to the total number of mononuclear cells present in a tissue.

*Expression of MHC class II*

In 16 of the 18 cases studied, HLA-DR positive cells were present in high densitites (score 3.5–4). The remaining two cases were scored with a moderate density of DR positive cells. In a previous work we had found that most of the tumour-associated mononuclear cells were T cells (CD3 +). Although B cells were not found in any of the 18 cases, macrophages comprised approximately one-third of the TAMC in a small number of cases. Thus, these macrophages would have accounted for some of the class II positive cells. However, using a dual immunoenzyme labelling system (Hohmann *et al.*, 1988) we demonstrated that all CD3 + cells expressed DR.

HLA-DP cells were found in high density in 14 of 18 cases (score 3–3.5) and in moderate density in the remaining four cases (score 1.5–2.0). Cells expressing DR and DP were present in all cases and neither their presence nor relative density showed any relation to tumour stage.

Although only four stage D tumours were available, three of these were HLA-DQ negative and were the only HLA-DQ negative cases of the 18 tumours examined. Although based on a small sample size, this finding may be of some significance since not only during ontogeny are the class II antigens differentially expressed (Edwards *et al.*, 1985) but cells expressing different class II gene products appear to play different roles in immune responses (Natali *et al.*, 1986). In particular, HLA-DQ + T cells have been reported to be important in effector functions (Navarrete *et al.*, 1986) and a DQw1 mAb has been shown to block the generation of cytotoxic T cells in a mixed-lymphocyte reaction (MLR) (Corte *et al.*, 1982).

In those three cases lacking HLA-DQ + cells, the infiltrating cells expressed other 'activation' markers and the total number of infiltrating cells, although reduced in number from that of stage A tumours, was not significantly different from stage B or C tumours where HLA-DQ + cells were readily found.

*Expression of IL2R*

Ralfkiaer *et al.* (1987) reported that most of the T lymphocyte infiltrate in malignant melanomas expressed the IL2R. However, in our study IL2R + TATL were present in only low density (score ⩽ 1) and only in four of the 18 cases. The lack of reactivity with the anti-IL2R mAb on its own could not rule out the presence of IL2R on the TATL. The anti-Tac mAb reacts only with the low affinity IL2R and the presence of the high affinity IL2R cannot be excluded using this mAb (Smith, 1988). Furthermore, interleukin 2 released by the TATL and bound to the IL2R on these cells may block the reactivity of this mAb (Leonard *et al.*, 1983).

*Expression of CD45*

In all cases cells expressing the p180 antigen were present while CD45R + (p220) cells were found in only five of the 18 cases. These five cases consisted of one stage A, one stage B, two stage C and one stage D case. From other works which have investigated the expression of the CD45 antigens on T cells it appears that cells progress from expression of the CD45R epitope to the p180 epitope as a consequence of activation and that p180 + cells represent a primed/memory subset of T cells (Akbar *et al.*, 1988; Serra *et al.*, 1988). If this hypothesis is correct, our findings indicate that the p180 + cells are primed or 'committed' effector cells within the tumour tissue. However, the predominance of p180 + cells may be due to the recruitment of these cells into the tumours. This is suggested by the work of Pitzalis *et al.* (1988), who showed that p180 + cells preferentially accumulated in inflammatory lesions. As p180 + and p220 + cells have distinct requirements for lymphokines (Greenbaum *et al.*, 1988), their recruitment may be influenced by the release of cytokines by other tumour-associated mononuclear cells.

In summary, it appears that most of the TATL in colonic carcinomas may be primed and activated T cells by virtue of their expression of the class II antigens and the p180 antigen. Conversely, there may have been a preferential infiltration of the distinct subset of CD4 +/UCHL1 + T cells into the tumour site. The lack of IL2R was unexpected. The absence of HLA – DQ + TATL only in stage D tumours suggests that HLA – DQ + may be an important marker for a type of T cell involved in effective tumour reactions and warrants further investigation in more late stage tumours.

Mr M.N. Norazmi is supported by the Universiti Sains Malaysia.

**References**

AKBAR, A.N., TERRY, L., TIMMS, A., BEVERLEY, P.C.L. & JANOSSY, G. (1988). Loss of CD45R and gain of UCHL1 reactivity is a feature of primed T cells. *J. Immunol.*, **140**, 2171.

CHEN, Y.X., EVANS, R.L., POLLACK, M.S. & 5 others (1984). Characterization and expression of the HLA-DC antigens defined by anti-Leu 10. *Hum. Immunol.*, **10**, 221.

CORTE, G., MORETTA, A., COSULICH, M.E., RAMARLI, D. & BARGELLESI, A. (1982). A monoclonal anti-DC1 antibody selectively inhibits the generation of effector T cells mediating specific cytolytic activity. *J. Exp. Med.*, **156**, 1539.

CSIBA, A., WHITWELL, H.L. & MOORE, M. (1984). Distribution of histocompatibility and leukocyte differentiation antigens in normal human colon and in benign and malignant colonic neoplasms. *Br. J. Cancer*, **50**, 699.

DAVIS, N.C. & NEWLAND, R.C. (1983). Terminology and classification of colorectal cancer: the Australian clinico-pathological staging system. *Aust. NZ. J. Surg.*, **53**, 211.

DOWER, S.K., HEFENEIDER, S.H., ALPERT, A.R. & URDAL, D.L. (1985). Quantitative measurement of human IL-2 receptor levels with intact and detergent solubilized human T cells. *Mol. Immunol.*, **22**, 937.

EDWARDS, J.A., JONES, D.B., EVANS, P.R. & SMITH, J.L. (1985). Differential expression of HLA Class II antigens on human foetal and adult lymphocytes and macrophages. *Immunology*, **55**, 489.

GREENBAUM, L.A., HOROWITZ, J.B., WOODS, A., PASQUALINI, T., REICH, E.-P. & BOTTOMLY, K. (1988). Autocrine growth of CD4 + cells: Differential effects of IL-1 on helper and imflammatory T cells. *J. Immunol.*, **140**, 1555.

HOHMANN, A., HODGSON, A.J., DI, W., SKINNER, J.M., BRADLEY, J. & ZOLA, H. (1988). Monoclonal alkaline phosphatase-anti-alkaline phosphatase (APAAP) complex: production of antibody, optimization of activity and use in immunostaining. *J. Histochem. Cytochem.*, **36**, 137.

HSU, S.-M., RAINE, L. & FANGER, H. (1981). Use of avidin-biotin-peroxidase complex (ABC) in immunoperoxidase techniques:a comparison between ABC and unlabelled antibody (PAP) procedures. *J. Histochem. Cytochem.*, **29**, 577.

LAMPSON, L.A. & LEVY, R. (1980). Two populations of Ia-like molecules on a human B cell line. *J. Immunol.*, **125**, 293.

LEDBETTER, J.A., EVANS, R.L., LIPINSKI, M., CUNNINGHAM-RUNDLES, C., GOOD, R.A. & HERZENBERG, L.A. (1981). Evolutionary conservation of surface molecules that distinguish T lymphocyte helper-inducer and T cytotoxic/suppressor subpopulations in mouse and man. *J. Exp. Med.*, **153**, 310.

LEONARD, W.J., DEPPER, J.M., ROBB, R.J., WALDMAN, T.A. & GREENE, W.C. (1983). Characterization of the human receptor for T-cell growth factor. *Proc. Natl Acad. Sci. USA.*, **80**, 6957.

NATALI, P., BIGOTTI, A., CAVALIERI, R. & 6 others (1986). Gene products of the HLA-D region in normal and malignant tissues of nonlymphoid origin. *Hum. Immunol.*, **15**, 220.

NAVARRETE, C., FERNANDEZ, N., ALONSO, M.C. & FESTENSTEIN, H. (1986). Ontogenic and functional implications of the differential expression of HLA-DQ antigens on leukaemic cells. *Hum. Immunol.*, **16**, 52.

PITZALIS, C., KINGSLEY, G., HASKARD, D. & PANAYI, G. (1988). The preferential accumulation of helper-inducer T lymphocytes in inflammatory lesions: evidence for regulation by selective endothelial and homotypic adhesion. *Eur J. Immunol.*, **18**, 1397.

RALFKIAER, E., HOU-JENSEN, K., GATTER, K.C., DRZEWIECKI, K.T. & MASON, D.Y. (1987). Immunohistological analysis of the lymphoid infiltrate in cutaneous malignant melanomas. *Virchows Arch. A*, **410**, 355.

ROWE, D.J. & BEVERLEY, P.C.L. (1984). Characterization of breast cancer infiltrates using monoclonal antibodies to human leukocyte antigens *Br. J. Cancer*, **49**, 149.

ROYSTON, I., OMARY, M.B. & TROWBRIDGE, I.S. (1981). Monoclonal antibodies to a human T-cell antigen and la-like antigen in the characterization of lymphoid leukaemias. *Transplant. Proc.*, **13**, 761.

SERRA, H.M., KROWKA, J.F., LEDBETTER, J.A. & PILARSKI, L.M. (1988). Loss of CD45R (Lp220) represents a post-thymic T cell differentiation event. *J. Immunol.*, **140**, 1435.

SMITH, S.H., BROWN, M.H., ROWE, D., COLLARD, R.E. & BEVERLEY, P.C.L. (1986). Functional subsets of human helper-inducer cells defined by a new monoclonal antibody, UCHL1. *Immunology*, **58**, 63.

SMITH, K.A. (1988). The bimolecular structure of the interleukin 2 receptor. *Immunol. Today*, **9**, 36.